# Association of Vitamin D Receptor Gene Polymorphisms and Hypovitaminosis D with Reduced Bone Mineral Density in Survivors of Childhood Leukemia: A Study in Algerian Patients

**DOI:** 10.3390/cimb48050506

**Published:** 2026-05-14

**Authors:** Wafa Khelaifia, Ines Gouaref, Fatma Zohra Djaballah-Ider, Nabila Bouterfas, Chafia Touil-Boukoffa, Assia Galleze

**Affiliations:** 1 Cytokines and Nitric Oxide Synthases Team, Cellular and Molecular Biology Laboratory, Faculty of Biological Sciences, Houari Boumediene University of Sciences and Technology, Algiers 16111, Algeria; 2 Bioenergetics and Intermediary Metabolism Team, Department of Biology and Organism Physiology, Faculty of Biological Sciences, Houari Boumediene University of Sciences and Technology, Algiers 16111, Algeria; 3 Pediatric Department, Beni Messous University Hospital, Algiers 16206, Algeria

**Keywords:** hypovitaminosis D, survivors of childhood leukemia, bone mineral density, VDR polymorphisms, PCR-RFLP

## Abstract

Survivors of childhood leukemia are at increased risk of long-term skeletal complications, including reduced bone mineral density (BMD). Vitamin D deficiency and genetic variations in the vitamin D receptor (VDR) gene are important factors influencing bone health, yet their combined effects remain insufficiently studied, particularly in North African populations. This case-control study included 130 survivors of childhood acute lymphoblastic leukemia (ALL) in remission (age range: 5–26 years) and 110 age- and sex-matched healthy controls recruited from Beni Messous Hospital. BMD was assessed at the lumbar spine and femoral neck using dual-energy X-ray absorptiometry and expressed as z-scores. Serum 25-hydroxyvitamin D levels were measured, and VDR polymorphisms (FokI, ApaI, and BsmI) were analyzed using PCR-RFLP. Hypovitaminosis D was observed in 43.85% of patients at diagnosis and 23.07% after remission. Survivors had significantly lower BMD compared with controls at both the lumbar spine (z-score: −4.26 ± 0.75 vs. 0 ± 1, *p* < 0.001) and femoral neck (−3.78 ± 0.45 vs. 0 ± 1, *p* < 0.001). Reduced BMD for age was identified in 30% of patients. Variant genotypes TT (FokI), AA (BsmI), and CC (ApaI) were more frequent in patients and were associated with lower BMD (*p* < 0.0001). These findings suggest that hypovitaminosis D and VDR polymorphisms may be associated with bone health in survivors of childhood leukemia. The coexistence of these factors may contribute to interindividual variability in BMD.

## 1. Introduction

Childhood leukemia represents the most common malignancy in pediatric populations, particularly acute lymphoblastic leukemia (ALL), which accounts for the majority of cases [1,2,3,4]. Advances in treatment strategies, including risk-adapted chemotherapy, targeted therapies, and improved supportive care, have significantly increased survival rates over recent decades, with five-year survival now exceeding 80–90% in high-income settings [5,6,7]. However, long-term survivors are increasingly recognized to be at risk for chronic complications, including metabolic and skeletal disorders [8]. Reduced bone mineral density (BMD) and increased fracture risk have been frequently reported in this population, largely due to the combined effects of chemotherapy, corticosteroid exposure, reduced physical activity, and endocrine disturbances [9,10].

Vitamin D plays a central role in bone metabolism by regulating calcium and phosphate homeostasis and promoting bone mineralization [11,12,13,14]. Hypovitaminosis D is highly prevalent among childhood cancer survivors and has been identified as a major modifiable risk factor for impaired bone health, with studies reporting deficiency rates ranging from 14% to over 35% in this population [15,16]. Recent clinical evidence further emphasizes that hypovitaminosis D remains common even after remission and contributes significantly to long-term skeletal complications, including reduced bone mineral density and increased fracture risk [17,18]. The biological effects of vitamin D are mediated through the vitamin D receptor (VDR), a nuclear transcription factor that regulates the expression of genes involved in calcium absorption, bone remodeling, and mineral metabolism [19,20,21,22]. Genetic variations in the VDR gene have been widely investigated as potential determinants of interindividual differences in bone mass and susceptibility to reduced BMD, with multiple studies demonstrating associations between VDR polymorphisms and bone mineral density as well as fracture risk [23,24]. Several polymorphisms, including FokI, BsmI, ApaI, and TaqI, have been associated with bone mineral density, fracture risk, and response to anti-osteoporotic therapies [25,26,27]. Moreover, VDR dysfunction has been shown to contribute to the pathogenesis of reduced BMD, highlighting its importance in skeletal homeostasis [28,29].

Although reduced bone mineral density and vitamin D deficiency have been well documented in survivors of childhood leukemia, relatively few studies have examined the contribution of genetic factors in this setting. Long-term skeletal complications, including impaired bone mineral density, are increasingly recognized among cancer survivors due to treatment-related and metabolic factors. However, studies specifically evaluating the role of genetic susceptibility, including VDR polymorphisms, in survivors of childhood leukemia remain limited.

Reduced BMD is a multifactorial condition resulting from the interaction between genetic and environmental factors. Evidence suggests that polymorphisms in the VDR gene can influence calcium metabolism and bone turnover, thereby modulating reduced BMD risk [30]. These genetic effects may vary across populations due to ethnic diversity, environmental exposures, and gene–environment interactions [31]. In particular, mutations or functional variations in the VDR gene may impair mineral metabolism and reduce bone density, increasing susceptibility to fractures [32].

Despite growing evidence linking hypovitaminosis D and VDR polymorphisms to reduced BMD, limited data are available in childhood leukemia survivors, especially in North African populations. Understanding the combined impact of hypovitaminosis D and genetic susceptibility is essential for improving risk stratification and developing targeted preventive strategies in this vulnerable group. Therefore, this study aimed to evaluate the association between vitamin D receptor gene polymorphisms and hypovitaminosis D with bone mineral density in survivors of childhood leukemia, and to explore their potential combined effects.

## 2. Materials and Methods

### 2.1. Study Subjects

In this case-control study, 130 patients treated for leukemia in Oncology Department of Beni Messous University Hospital were recruited from 2022 to 2026. As inclusion criteria, they were all in remission and their treatment had been completed at least one year earlier. Exclusion criteria included history of bone marrow transplantation, any secondary malignancy, hormone replacement therapy or chronic illnesses that affect bone metabolism. The control subjects were hospital-based volunteers recruited from individuals undergoing routine health examinations at the same medical institution as the patient group. They were selected to match the cases in terms of age, sex, BMI, physical activity, sun exposure, and nutritional status. Briefly, age matching was performed within comparable age categories, and BMI was matched within clinically comparable ranges. Physical activity, sun exposure, and nutritional status were assessed during clinical evaluation and participant interviews. Nutritional status was then supported by BMI evaluation. All controls were screened through clinical evaluation and medical history to confirm the absence of malignancy, chronic disease, or conditions affecting bone metabolism. This study has been approved by the local ethics committee, the “Algerian National Agency for Research in Health Sciences, ATRSS ex-ANDRS”, in compliance with the Helsinki declaration (Code number 58-DFPR-ATRSS-AAP-2014 and approved date 29 April 2018).

### 2.2. Evaluation of BMD

BMD (g/cm^2^) was evaluated using dual-energy X-ray absorptiometry (DEXA) on the lumbar spine and femoral neck. Reference data of pediatric BMD was obtained from the manufacturer and expressed in age- and gender-matched z-scores. In accordance with pediatric guidelines from the International Society for Clinical Densitometry, low BMD for age was defined as a z-score ≤ −2.0 [33].

### 2.3. 25-(OH)D Measurement

Serum 25-hydroxyvitamin D [25(OH)D] levels were measured using a chemiluminescent immunoassay (Elecsys Vitamin D Total; Roche Diagnostics, Mannheim, Germany) in the same laboratory according to standardized procedures. Vitamin D levels at diagnosis were measured prior to treatment initiation, while follow-up measurements were obtained at the time of study inclusion during remission. Blood samples were collected under fasting conditions, and the timing of sampling was recorded to account for potential seasonal variation in vitamin D levels. Information on vitamin D supplementation at the time of sampling was also recorded. Serum 25(OH)D concentrations were classified according to the Endocrine Society Clinical Practice Guidelines as deficiency (<20 ng/mL), insufficiency (21–29 ng/mL), and sufficient (≥30 ng/mL).

### 2.4. Genetic Analyses

Genomic DNA was extracted from whole blood using the PureLink^®^ Genomic DNA Extraction Kit (Thermo Fisher Scientific, Waltham, MA, USA). Vitamin D receptor (VDR) gene polymorphisms (FokI, ApaI, and BsmI) were analyzed using the polymerase chain reaction restriction fragment length polymorphism (PCR-RFLP) method with specific primers (Table 1). PCR amplification was performed in a total reaction volume of 25 μL containing 12.5 μL of master mix (including Taq polymerase and deoxynucleotide triphosphates), 2 μL of genomic DNA, 2 μL of each primer (forward and reverse), and 6.5 μL of distilled water. The thermal cycling conditions consisted of an initial denaturation at 95 °C for 5 min, followed by 40 cycles of denaturation at 95 °C for 15 s, annealing at 72 °C for FokI and 60 °C for BsmI for 60 s, and extension at 72 °C for 45 s. The amplified PCR products were digested with the following restriction enzymes according to the manufacturer’s instructions: *FokI*, *BsmI*, and *ApaI* restriction enzymes (Thermo Fisher Scientific, Waltham, MA, USA) and separated on a 2.5% agarose gel, then visualized under UV transillumination.

### 2.5. Statistical Analysis

Statistical analyses were performed using GraphPad Prism 8.0. Continuous variables were expressed as median (interquartile range). Normality was assessed using the Shapiro–Wilk test, and homogeneity of variance was evaluated using Levene’s test. Between-group comparisons were initially performed using Student’s *t*-test or one-way ANOVA for normally distributed variables. Categorical variables were compared using the chi-square test. To evaluate independent associations with bone mineral density (BMD), multivariable linear regression models were constructed, adjusting for potential confounders including body mass index (BMI) and remission duration. Logistic regression analysis was used to assess factors associated with low BMD (z-score ≤ −2.0), and odds ratios (ORs) with 95% confidence intervals (CIs) were reported. Correlation analyses were performed using Pearson coefficients. Comparisons of vitamin D levels at diagnosis and during remission were conducted. Bonferroni correction was applied for multiple comparisons involving vitamin D categories/genotype comparisons. A *p*-value < 0.05 was considered statistically significant.

## 3. Results

### 3.1. Patient Characteristics

The clinical characteristics of patients are shown in Table 2. The cohort consisted of patients with acute lymphoblastic leukemia, treated according to EORTC 2014 protocol Table 3. The median duration of remission was 6 years (IQR: 4–10). Among these patients, 56.15% were male and 43.84% were female with a sex ratio of 1.28. Mean BMI of patients was 18.41 ± 3.55, among which 58.48% were underweight.

### 3.2. Biochemical Analysis

**Figure 1:**
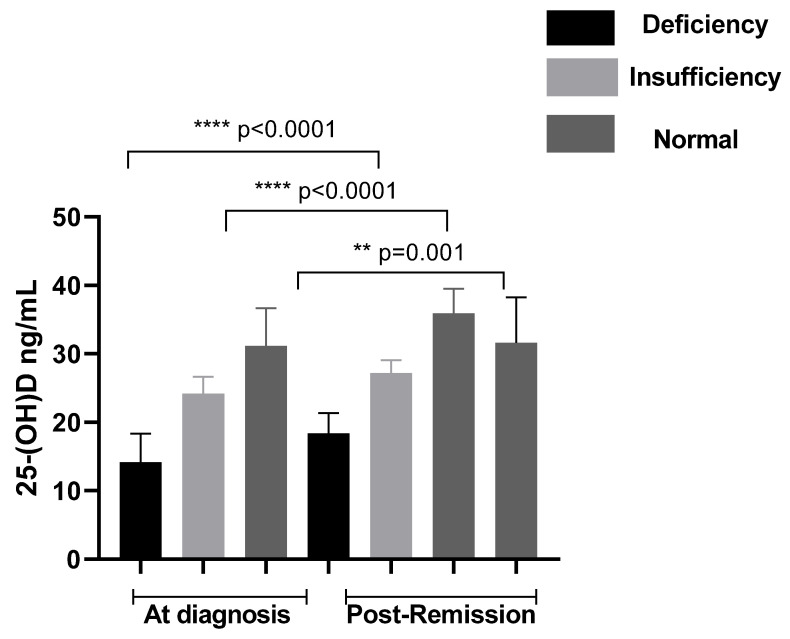
Vitamin D levels in patients at diagnosis and post-remission. Significant differences were considered for *p* < 0.05 (**** *p* ≤ 0.0001).

**Figure 2:**
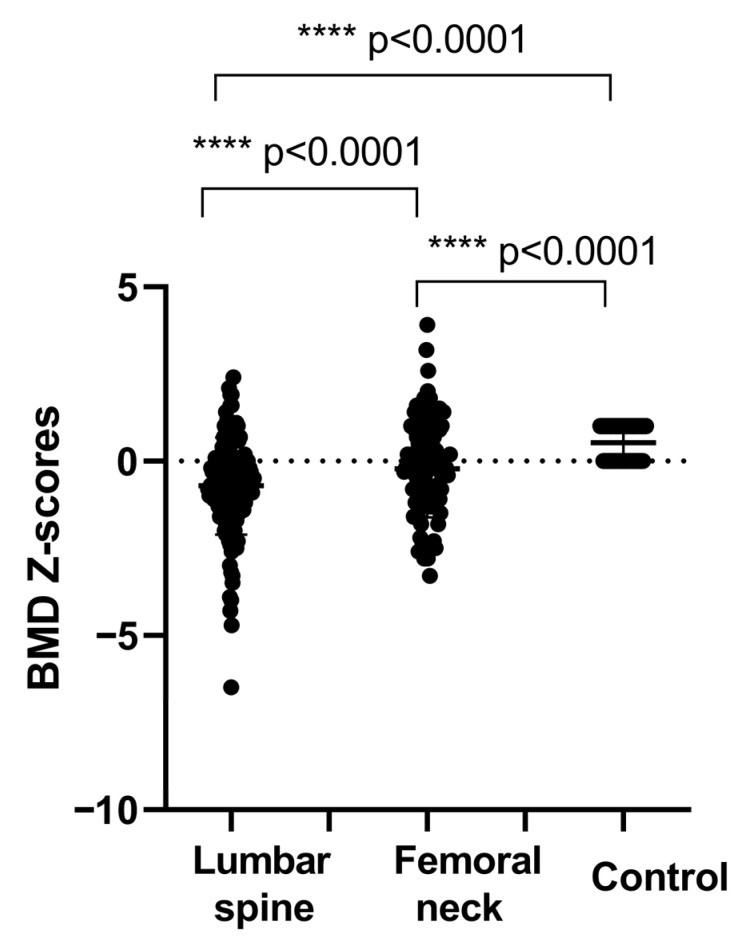
BMD z-scores in patients. Results are expressed as the mean ± SD. Significant differences were considered for *p* < 0.05 (**** *p* ≤ 0.0001).

Our data showed that at diagnosis, 43.85% of patients were vitamin D deficient (<20 ng/mL), while 50.0% exhibited insufficient levels (<30 ng/mL). A total of 51.53% of patients had vitamin D levels below 30 ng/mL during remission, including 28.46% with insufficiency and 23.07% with deficiency (Figure 1). In comparison, the control group showed a lower prevalence of vitamin D deficiency and insufficiency (15%), indicating a significantly poorer vitamin D status among patients. Mean BMD z-scores (±SD) at the lumbar spine (LS) and femoral neck (FN) were −4.26 ± 0.75 and −3.78 ± 0.45, respectively, and were significantly lower compared to controls (*p* < 0.001). Based on BMD classification, a proportion of patients exhibited reduced bone mineral density compared with controls. The distribution of BMD categories at LS and FN is presented in Figure 2. In addition, the results showed that body mass index correlated significantly with LS BMD (r = 0.45, *p* < 0.001) and FN BMD (r = 0.50, *p* < 0.001). Higher BMI was significantly associated with higher BMD and lower prevalence of reduced BMD in the studied population. The study showed also that there is a highly significant difference of BMD between patients according to duration of remission (*p* < 0.001).

### 3.3. Genotypic and Allelic Analysis

The distribution of vitamin D receptor genotypes and alleles among childhood leukemia survivors and controls is presented in Table 4. All polymorphisms were in Hardy-Weinberg equilibrium (ApaI: *p* = 0.210; BsmI: *p* = 0.415; FokI: *p* = 0.314). A significant difference in the genotypic distribution of VDR polymorphism was observed between patients and control group (*p* < 0.001). For the FokI polymorphism (rs2228570), frequency of TT genotypes was higher in patients compared to controls (57.69% vs. 21.82%, *p* < 0.0001). For the BsmI polymorphism (rs1544410), the distribution of GG, GA, and AA genotypes differed between groups, with a higher frequency of the variant genotype AA observed in patients compared to controls (50% vs. 18.18%, *p* < 0.0001). Similarly, for the ApaI polymorphism (rs7975232), the CC genotype was significantly more frequent in patients than in controls (61.54% vs. 14.55%, *p* < 0.0001). In contrast, the CC, GG and AA genotypes were significantly more prevalent in the control group than in patients (53.64% vs. 7.69%, *p* < 0.0001; 50.91% vs. 7.69%, *p* < 0.0001; 47.27% vs. 6.15%, *p* < 0.0001, respectively).

Allelic distribution analysis showed a significantly higher frequency of the T (FokI), A (BsmI), and C (ApaI) alleles in patients compared to controls. The T allele of FokI was more frequent in patients than in controls (75.0% vs. 34.1%; OR = 5.80, 95% CI: 3.89–8.65). Likewise, the A allele of BsmI was more common in patients (71.2% vs. 33.6%; OR = 4.82, 95% CI: 3.24–7.17), and the C allele of ApaI was overrepresented in patients compared with controls (77.7% vs. 33.6%; OR = 6.87, 95% CI: 4.53–10.43).

Under the dominant and recessive models, all three polymorphisms remained significantly associated with case status. The dominant models yielded ORs of 13.88 for FokI, 12.44 for BsmI, and 13.66 for ApaI, while the recessive models showed ORs of 4.89, 4.50, and 9.40, respectively. These findings support a significant association between VDR polymorphisms and disease susceptibility in the studied population. Adjusted odds ratios were obtained from multivariable logistic regression models including BMI and remission duration as covariates.

**Figure 3:**
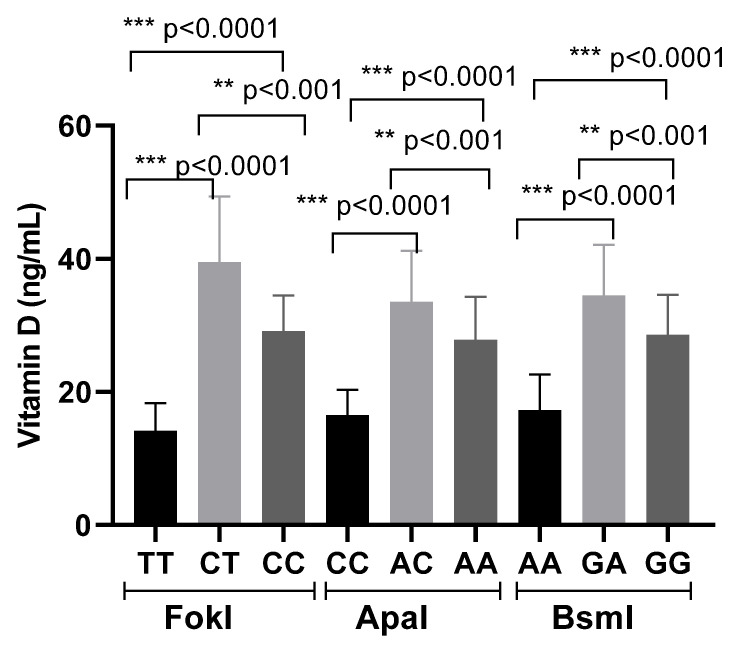
Vitamin D levels in post remission patients according to genotypes. Results are expressed as the mean ± SD. Significant differences were considered for *p* < 0.05 (*** *p* ≤ 0.001).

Furthermore, variations in vitamin D levels appeared to be influenced by VDR genotypes: patients carrying the heterozygous genotypes showed a mean increase of 15 ng/mL in serum 25(OH)D levels, whereas individuals with the homozygous variant genotypes exhibited a slight decrease of 5 ng/mL (Figure 3).

**Figure 4:**
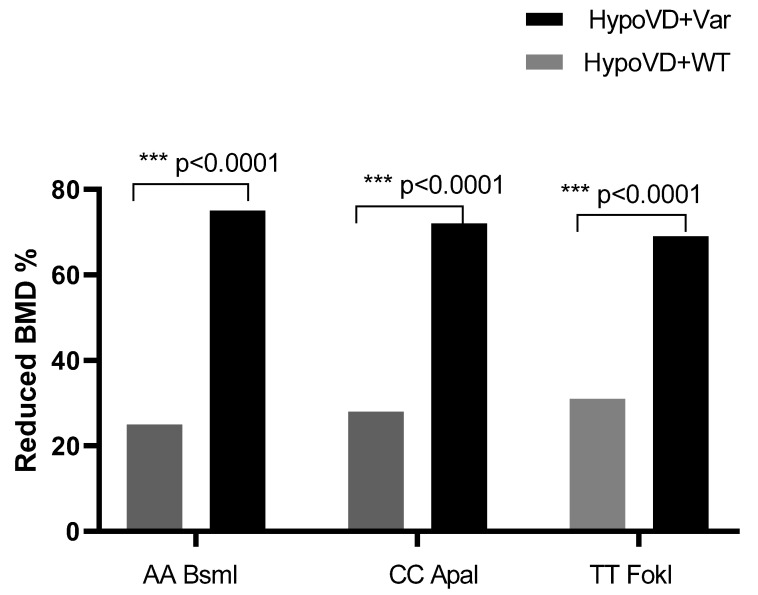
Distribution of Reduced Bone Mineral Density According to VDR Genotype and Vitamin D Status. Significant differences were considered for *p* < 0.05 (*** *p* ≤ 0.001). WT: wild-type genotype; Var: variant genotype.

When stratified according to bone status, variant genotypes particularly AA for BsmI, CC for ApaI and TT for FokI were more frequently observed in patients with reduced BMD compared to those with normal bone status (*p* < 0.0001) (Figure 4).

**Figure 5:**
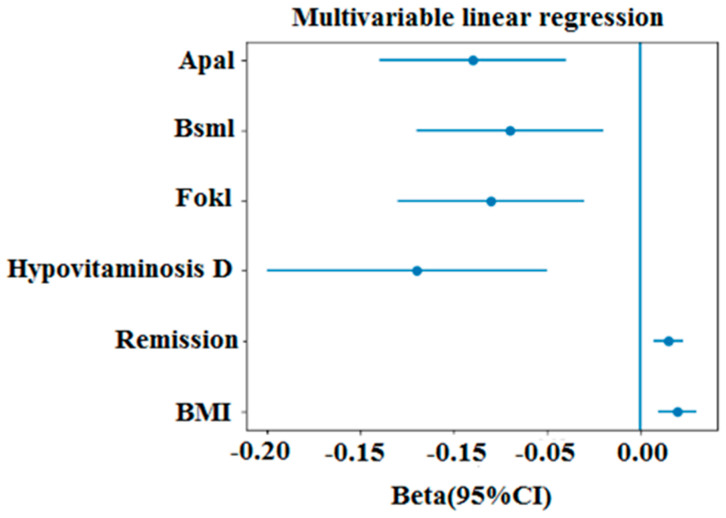
Multivariable linear regression analysis of factors associated with bone mineral density.

To identify independent predictors of bone mineral density, multivariable linear regression analyses were performed including body mass index, remission duration, vitamin D status, and VDR polymorphisms as covariates. BMI was independently and positively associated with BMD at both the lumbar spine (*p* < 0.001) and femoral neck (*p* < 0.001). Remission duration also showed a significant association with BMD (*p* < 0.001), suggesting that bone status may evolve over time following treatment. Hypovitaminosis D was associated with lower BMD values compared with sufficient levels (*p* < 0.01) (Table 5). Regarding genetic factors, variant genotypes of the VDR polymorphisms (FokI TT, BsmI AA, and ApaI CC) were associated with reduced BMD compared to wild-type genotypes in adjusted models (all *p* < 0.05) (Figure 5).

## 4. Discussion

Vitamin D mediates its biological effects primarily through the vitamin D receptor, a nuclear transcription factor that regulates the expression of genes involved in key cellular processes, including proliferation, differentiation, apoptosis, and metastasis. VDR signaling is known to interact with major intracellular pathways such as MAPK and PI3K/Akt, thereby modulating cellular growth, survival, and differentiation [34,35,36]. Accumulating evidence indicates that the anticancer properties of vitamin D are largely mediated through the suppression of malignant cell proliferation and the promotion of cellular differentiation. These effects are associated with the upregulation of cell cycle regulators, including p21 and p27, resulting in G0/G1 cell-cycle arrest and enhanced differentiation of tumor cells [37,38].

These mechanisms support the potential therapeutic role of vitamin D, VDR, and their analogues as differentiation-inducing agents in hematological malignancies. Recent studies have highlighted the relevance of vitamin D signaling in leukemia and lymphoma, where it may influence disease progression and treatment response [39,40]. Clinically, higher serum vitamin D levels in patients with acute myeloid leukemia (AML) have been associated with improved treatment response, longer survival, and better overall prognosis, whereas hypovitaminosis D has been consistently linked to poorer outcomes [41,42]. Furthermore, low vitamin D levels have been proposed as a prognostic biomarker in several hematological malignancies, including acute lymphoblastic leukemia (ALL), chronic lymphocytic leukemia (CLL), small lymphocytic lymphoma (SLL), T-cell lymphoma, and multiple myeloma, where deficiency is associated with reduced overall survival (OS) and increased disease severity [43,44].

Our findings are consistent with previous studies demonstrating reduced bone mineral density (BMD) in survivors of childhood acute lymphoblastic leukemia (ALL), a complication largely attributed to treatment-related factors such as prolonged corticosteroid exposure, reduced physical activity, and endocrine disturbances. Long-term skeletal complications are increasingly recognized in this population [45,46]. In parallel, we observed a high prevalence of vitamin D deficiency, which persisted, although to a lesser extent, after remission, in line with prior reports highlighting long-term alterations in vitamin D status among cancer survivors [47]. Given the well-established role of vitamin D in bone metabolism, this deficiency likely contributes to impaired bone health.

The association between VDR polymorphisms and bone mineral density has been widely investigated; however, findings remain inconsistent across different populations. Several studies in Asian populations have reported significant associations between FokI, BsmI, and ApaI polymorphisms and reduced BMD or increased osteoporosis risk, whereas studies in European cohorts have yielded mixed or even negative results. Similarly, investigations in Middle Eastern and North African populations have demonstrated variable genotype distributions and differing strengths of association with bone-related outcomes. These discrepancies may be explained by ethnic differences in allele frequencies, genetic background, and environmental influences such as sunlight exposure, dietary calcium intake, and vitamin D status. In this context, the relatively strong associations observed in the present Algerian cohort may reflect population-specific genetic susceptibility.

In our cohort, markedly reduced BMD values were observed. The positive correlation between BMI and BMD further underscores the importance of nutritional and metabolic factors, while the association between remission duration and BMD suggests that bone status may evolve over time following treatment. The observed differences in VDR polymorphism distribution between patients and controls suggest a possible association between genetic factors and bone health. Previous studies have reported associations between VDR gene variants and BMD, fracture risk, and therapeutic response, although findings remain inconsistent across populations [48,49,50]. In our study, the coexistence of vitamin D deficiency and specific VDR genotypes was associated with lower BMD, suggesting a possible combined effect of environmental and genetic factors.

However, this study has several limitations that should be considered when interpreting the findings. First, the case–control design precludes causal inference and limits the ability to establish temporal relationships. Second, although controls were included, potential differences in baseline characteristics may affect comparability between groups. Third, the relatively wide age range of participants may introduce heterogeneity, particularly given the age-dependent variation in bone mineral density and vitamin D metabolism. In addition, the sample size, especially for genetic analyses, was modest and may limit statistical power. Although associations between VDR polymorphisms and reduced BMD were observed, formal gene–environment interaction analyses were not performed, and therefore the combined effects of genetic and environmental factors (such as vitamin D status) remain unclear. Furthermore, residual confounding from unmeasured factors, including nutritional intake, physical activity, hormonal status, and treatment-related variables, cannot be excluded. Finally, these findings require confirmation in larger, prospective, and ideally multiethnic studies to better clarify the role of VDR polymorphisms in bone health among survivors of childhood leukemia.

## 5. Conclusions

In this case-control study, survivors of childhood leukemia showed a high prevalence of hypovitaminosis D and reduced bone mineral density compared with controls. VDR polymorphisms were also differently distributed between patients and controls and were associated with lower BMD. The coexistence of hypovitaminosis D and selected VDR genotypes may indicate a possible combined effect on bone health. These results suggest that vitamin D status and VDR genetic variation may be associated with skeletal health in survivors of childhood leukemia. Further prospective studies with larger cohorts, age-stratified analyses, detailed treatment exposure data, and adjustment for nutritional, hormonal, lifestyle, and clinical confounders are needed to confirm these findings.

## Figures and Tables

**Table 1:** Primers used for VDR gene polymorphism analysis.

Polymorphism	Forward Primer (5′ → 3′)	Reverse Primer (5′ → 3′)
**FokI**	AGCTGGCCCTGCACTGACTCTGCTCT	ATGGAAACACCTTGCTTCTTCTCCCTC
**BsmI**	CAACCAAGACTACAAGTACCGCGTCAGTGA	AACCAGCGGGAAGAGGTCAAGGG
**ApaI**	CAGAGCATGGACAGGGAGCAA	TCATGGCTGAGGTCTCAAGGG

**Table 2:** Patient characteristics.

Characteristics	Patients (N = 130)	Controls (N = 110)
**Gender**		
Male	73 (56.15)	58 (52.72)
Female	57 (43.84)	52 (47.27)
**Age**		
Mean	13	12
Range (years)	5–26	6–24
BMI (kg/m^2^)	18.41 ± 3.55	19.1 ± 3.25
**Vitamin D level**		
**Vitamin D status**		
Normal	63 (48.47)	93 (84.54)
Deficiency	30 (23.07)	4 (3.64)
Insufficiency	37 (28.46)	13 (11.82)
Normal	31.13 ± 5.54	31.59 ± 6.68
Deficiency	14.16 ± 4.17	17.28 ± 5.12
Insufficiency	24.16 ± 2.48	25.05 ± 3.18
**Leukemia subtype**		
Acute lymphoblastic leukemia	130	-
Remission duration	6 (4–10)	-

**Table 3:** Doses and duration of drug treatment.

Day 8	Day 12	Day 15	Day 22	Day 25	Day 29
Prednisone (60 mg/m^2^)		Prednisone (60 mg/m^2^)	Prednisone (60 mg/m^2^)	Prednisone (60 mg/m^2^)	Prednisone (60 mg/m^2^)
Vincristine (1.5 mg/m^2^)		Vincristine (1.5 mg/m^2^)	Vincristine (1.5 mg/m^2^)		Vincristine (1.5 mg/m^2^),
Doxorubicin (30 mg/m^2^)		Doxorubicin (30 mg/m^2^)	Doxorubicin (30 mg/m^2^)		Doxorubicin (30 mg/m^2^)
		PEG-Asp (2500 IU/m^2^)		PEG-Asp (2500 IU/m^2^)	
	IT Ara-C (30 mg)	IT MTX (15 mg)		IT MTX (15 mg)	

**Table 4:** Genotype frequencies of ApaI, Bms I, and FokI in patients and controls.

Genotypes	Patients (N = 130)	*p*	OR (95% CI)	Control (N = 110)
**FokI**				
**TT**	**75 (57.69%)**	**1.1 × 10^−8^**	**4.89 (2.67–9.05)**	**24 (21.82%)**
CT	45 (34.62%)	0.06	1.63 (0.89–2.99)	27 (24.55%)
**CC**	**10 (7.69%)**	**1.1 × 10^−15^**	**0.07 (0.03–0.16)**	**59 (53.64%)**
**BsmI**				
**AA**	**65 (50.0%)**	**1.8 × 10^−7^**	**4.5 (2.49–8.24)**	**20 (18.18%)**
GA	55 (42.31%)	0.06	1.64 (0.96–2.81)	34 (30.91%)
**GG**	**10 (7.69%)**	**2.4 × 10^14^**	**0.08 (0.03–0.18)**	**56 (50.91%)**
**ApaI**				
**CC**	**80 (61.54%)**	**3.1 × 10^−14^**	**9.40 (4.79–18.95)**	**16 (14.55%)**
AC	42 (32.31%)	0.20	0.77 (0.44–1.36)	42 (38.18%)
**AA**	**8 (6.15%)**	**6 × 10^−14^**	**0.07 (0.03–0.17)**	**52 (47.27%)**

**Table 5:** Multivariable linear regression analysis of factors associated with BMD.

Variable	Lumbar Spine BMD (β, 95% CI)	*p*	Femoral Neck BMD (β, 95% CI)	*p*
**BMI (kg/m^2^)**	0.019 0.009–0.029)	<0.001	0.017 (0.008–0.027)	<0.001
**Remission duration**	0.017 (0.010–0.025)	<0.001	0.013 (0.006–0.020)	<0.001
**Hypovitaminosis D**	0.027 (0.010–0.032)	<0.01	0.022 (0.010–0.026)	<0.01
**FokI (TT vs. CT + CC)**	−0.080 (−0.140–−0.030)	<0.0001	−0.070 (−0.130–−0.020)	<0.0001
**BsmI (AA vs. GA + GG)**	−0.072 (−0.130–−0.020)	<0.0001	−0.060 (−0.120–−0.010)	<0.0001
**ApaI (CC vs. AC + AA)**	−0.090 (−0.150–−0.040)	<0.0001	−0.080 (−0.140–−0.030)	<0.0001

## Data Availability

The original contributions presented in this study are included in the article. Further inquiries can be directed to the corresponding author.
